# pH-activated, mitochondria-targeted, and redox-responsive delivery of paclitaxel nanomicelles to overcome drug resistance and suppress metastasis in lung cancer

**DOI:** 10.1186/s12951-021-00895-4

**Published:** 2021-05-22

**Authors:** He Wang, Wenwen Shi, Danning Zeng, Qiudi Huang, Jiacui Xie, Huaying Wen, Jinfang Li, Xiyong Yu, Linghao Qin, Yi Zhou

**Affiliations:** 1 The Fifth Affiliated Hospital, Key Laboratory of Molecular Target and Clinical Pharmacology and the State Key Laboratory of Respiratory Disease, School of Pharmaceutical Sciences, Guangzhou Medical University, Guangdong 511436 Guangzhou, People’s Republic of China; 2 Center of Cancer Research, The Second Affiliated Hospital, Guangzhou Medical University, Guangdong 510260 Guangzhou, People’s Republic of China; 3 School of Pharmacy, Guangdong Pharmaceutical University, Guangzhou, 510006 Guangdong People’s Republic of China; 4 Department of Pharmaceutical Sciences, Xinjiang Second Medical College, Kelamayi, 830011 Xinjiang People’s Republic of China

**Keywords:** pH-Activated Mitochondria-Targeted Delivery, Redox-responsive, Lung cancer, Drug resistance, Metastasis

## Abstract

**Background:**

Mitochondria play a role in the occurrence, development, drug resistance, metastasis, and other functions of cancer and thus are a drug target. An acid-activated mitochondria-targeting drug nanocarrier with redox-responsive function was constructed in the present study. However, whether this vector can precisely delivery paclitaxel (PTX) to enhance therapeutic efficacy in drug-resistant lung cancer is unknown.

**Results:**

Acid-cleavable dimethylmaleic anhydride (DA) was used to modify pluronic P85-conjugated mitochondria-targeting triphenylphosphonium (TPP) using disulfide bonds as intermediate linkers (DA-P85-SS-TPP and DA-P-SS-T). The constructed nanocarriers demonstrated enhanced cellular uptake and selective mitochondrial targeting at extracellular pH characteristic for a tumor (6.5) and were characterized by extended circulation in the blood. TPP promoted the targeting of the DA-P-SS-T/PTX nanomicelles to the mitochondrial outer membrane to decrease the membrane potential and ATP level, resulting in inhibition of P-glycoprotein and suppression of drug resistance and cancer metastasis. PTX was also rapidly released in the presence of high glutathione (GSH) levels and directly diffused into the mitochondria, resulting in apoptosis of drug-resistant lung cancer cells.

**Conclusions:**

These promising results indicated that acid-activated mitochondria-targeting and redox-responsive nanomicelles potentially represent a significant advancement in cancer treatment.

**Graphic Abstarct:**

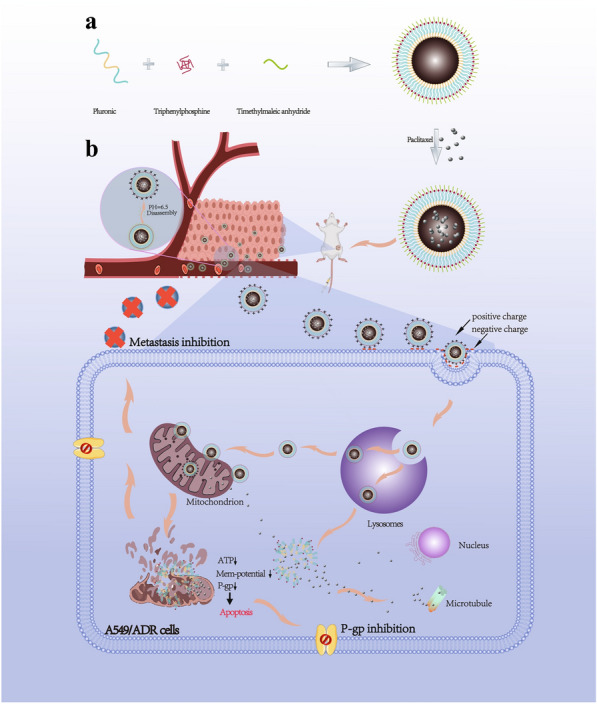

## Introduction

Lung cancer is malignant tumor, accounting for rapidly increasing morbidity and mortality and representing the biggest threat to the health and life of the population [1]. Despite recent treatment advances, multidrug resistance (MDR) remains a major challenge in lung cancer treatment [2]. MDR is caused by multiple factors and is mediated by various intrinsic and acquired mechanisms [3]. Mitochondria are the power source of the cells, play an important role in energy generation, apoptosis, signal transduction, cell cycle, and cell differentiation, and are closely related to carcinogenesis [4]. Multiple resistance mechanisms associated with abnormal mitochondrial activity have been reported [5–7]. MDR cells are characterized by a larger mitochondrial cluster and higher mitochondrial polarization compared with those in non-MDR cells [7]. MDR is caused by overexpression of the drug efflux pumps, which require ATP from the mitochondria; thus, mitochondrial targeting is a particularly interesting option for the treatment of drug-resistant cancer [8]. Tumor metastasis is another challenging aspect of cancer treatment and remains the leading cause of cancer-related mortality [9]. Cancer progression and metastasis indirectly or directly result from ATP, which is closely linked to the mitochondria [10]. Thus, disruption of the energy supply by targeted delivery of the drugs to the mitochondria may be an effective alternative to overcome drug resistance and cancer metastasis [11].

However, blocking the energy supply by targeting the mitochondria is difficult. The mitochondrial membrane is thick, has high negative potential, and is characterized by hydrophobicity and dense double-membrane structure that acts as a strong barrier preventing the entry of bioactive compounds [12]. Thus, the therapeutic effects of mitochondria-damaging anticancer drugs can be extremely limited. Paclitaxel (PTX) is one of these drugs commonly used as a first-line treatment of lung cancer and active against a variety of cancers. The effects of PTX mainly involve mitochondria [13] and microtubules [14] of cancer cells. Selective delivery of a drug to the mitochondria can be achieved by mitochondria-targeting signal peptides [15], oligoguanidinium [16], and triphenylphosphonium (TPP) [17] to modify drug carriers in the drug delivery systems. TPP is often used in delocalized lipophilic cations, which are usually modified on the surface of nanomicelles or covalently linked with nanocarriers to achieve mitochondrial targeting [3]. Our previous study demonstrated that TPP-modified pluronic F127 (PF127), which was decorated by hyaluronic acid (HA) as a vector (TPP-PF127-HA), was able to transfer PTX to the mitochondria and disrupt their function [18]; however, PF127 has a high hydrophilic-lipophilic balance and does not significantly reverse MDR in cancer cells [19].

**Scheme 1:**
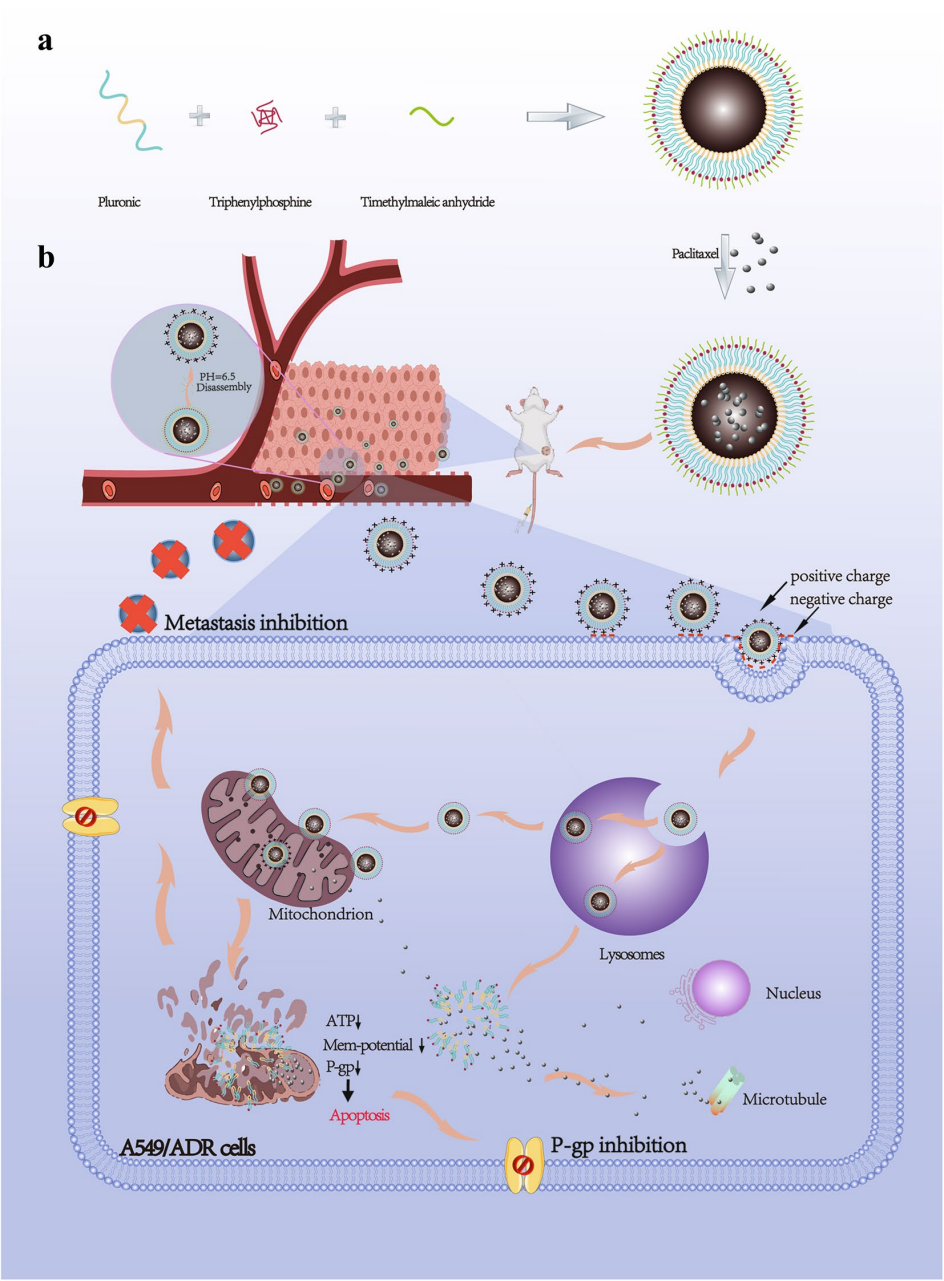
Scheme of the structures and proposed mechanism of action of DA-P-SS-T/PTX to overcome drug resistance and lung cancer metastasis. **A** Synthesis of DA-P-SS-T and fabrication scheme of DA-P-SS-T/PTX nanomicelles. **B** Scheme of the transport pathway after intravenous injection of DA-P-SS-T/PTX nanomicelles

Pluronic P85 (P85) was shown to reverse drug resistance in a number of studies [20, 21] and was thus used to replace PF127 in the present study to suppress MDR in lung cancer. Unlike TPP-PF127-HA, P85 was conjugated with TPP via an intermediate linker of disulfide bonds (P85-SS-TPP, P-SS-T) to enable quick release of the drug in cancer cells. Additionally, acid-cleavable dimethylmaleic anhydride (DA) protection group was used to modify P-SS-T to extend its circulation in the blood and increase endocytosis into the tumor cells. Acidic extracellular environment of the tumor with pH (≈ 6.5) induced hydrolysis of amide bonds and accelerated the internalization of the nanoparticles with selective restoration of the mitochondria-targeting ability of TPP [22]. After accumulation in the mitochondria, the released PTX induced mitochondrial dysfunction to inhibit the energy supply and decrease the expression of P-gp-related proteins and hindered metastasis; glutathione (GSH)-triggered redox responsiveness contributed to the disassembly of the nanoparticles eventually resulting in the release of PTX that directly diffused to act on microtubule proteins. Thus, we developed an acid-activated, charge-reversing, and redox-responsive drug nanocarrier for precise mitochondria-targeting delivery of PTX to reverse drug resistance and inhibit metastasis of lung cancer (Scheme 1).

## Materials and methods

### Materials

Pluronic P85 (M.W. = 4600) was obtained from BASF Aktiengesellschaft, and (5-carboxypentyl)triphenylphosphonium bromide (TPP-COOH) was provided by Bailingwei Co., Ltd. (Guangzhou, China). GSH (reduced) and DA were provided by Energy Chemical Co., Ltd. (Shanghai, China). Bis(2-hydroxyethyl) disulfide was obtained from TCI Development Co., Ltd. (Shanghai, China). N,N′-Dicyclohexylcarbodiimide (DCC), 4-dimethylaminopyridine (DMAP), genistein, chlorpromazine, amiloride, coumarin 6 (C6), and PTX were supplied by Sigma-Aldrich (Shanghai, China). Penicillin-streptomycin, fetal bovine serum (FBS), and RPMI 1640 were purchased from Gibco Life Technologies (USA). An ATP assay kit was provided by Beyotime Biotechnology. MitoTracker Red and LysoTracker® Red were supplied by Yeasen Biotech Co., Ltd. (Shanghai, China). All other reagents and solvents were of analytical grade and were used without further purification.

### Synthesis of Boc-P85-COOH

Boc-P85 was synthesized according to the following procedure. In brief, P85 (4.6 g, 1 mmol) and thionyl chloride (0.12 g, 1 mmol) were dissolved in 20 mL of dichloromethane (DCM) and stirred at 55 °C for 4 h. Ammonium hydroxide was dropwise added at room temperature. Then, di-tert-butyl dicarbonate ((Boc)_2_O) (0.22 g, 1 mmol) solution was dropwise added into the reaction within 8 h, and incubation was continued for another 24 h; then, the reaction mixture was filtered. White Boc-P85 product was obtained by drying under vacuum (Boc-P85, 71% yield).

Boc-P85-COOH was synthesized according to a previously reported protocol [23]. Briefly, 1,8-diazabicycloundec-7-ene (DBU; 4.6 mL, 3 mmol) was slowly added to a mixture of Boc-P85 (4.1 g, 1 mmol) and succinic anhydride (300.3 mg, 3 mmol) in 30 mL of dichloromethane at 0 °C. The reaction mixture was stirred at room temperature for 4 h, quenched with water (20 mL), and acidified with an aqueous HCl solution (1%). The yellow precipitate was collected and washed with aqueous HCl solution (1%, 15 mL× 3) and H_2_O (15 mL× 3) (Boc-P85-COOH, 78%).

### Synthesis of Boc-P85-SS-TPP

Boc-NH-SS-NH_2_ was synthesized according to a previously reported protocol [24]. The following protocol was used for the synthesis of Boc-NH-SS-TPP. In brief, Boc-NH-SS-NH_2_ (0.285 g) was dissolved under stirring in 10 mL of DMF containing 0.5 mL of trifluoroacetic acid (TFA). Subsequently, TPP-COOH (0.31 g, 1 mmol), DCC (0.50 g, 2.4 mmol), and DMAP (0.2 g, 1.70 mmol) were added to the solution and stirred at room temperature for 48 h. The reaction solution was filtered and concentrated under vacuum to obtain the dry product (Boc-NH-SS-TPP, 76% yield).

Boc-P85-SS-TPP was synthesized according to the following procedure. Boc-NH-SS-TPP (0.56 g) was dissolved under stirring in 10 mL of DCM containing 0.2 mL of TFA. Boc-P85-COOH (4.2 g, 1 mmol), DMAP (0.12 g, 1 mmol), and DCC (0.74 g, 3.6) were then dropwise added to the reaction solution at room temperature, and the incubation was continued for 24 h. The reaction solution was filtered and concentrated under vacuum to obtain the final dry product (Boc-P85-SS-TPP, 62% yield).

### Synthesis of DA-P85-SS-TPP

Boc-P85-SS-TPP (2 g) was dissolved under stirring in 20 mL of DMF containing 1 mL of TFA. Subsequently, DA (198 mg, 2 mmol) was added to the solution and stirred at room temperature for 48 h. The reaction solution was purified by dialysis (MWCO 1,000) against deionized water for 3 days and lyophilized to obtain DA-P85-SS-TPP (DA-P-SS-T, 60%).

### Preparation of DA-P-SS-T/PTX nanomicelles

PTX-loaded nanomicelles were prepared by nanoprecipitation as described previously [18]. In brief, DA-P-SS-T and PTX were dissolved in double-distilled water and ethanol, respectively. Then, PTX solution was dropwise added into the DA-P-SS-T solution under stirring for 1 h. The solution was dialyzed (MWCO 1000) against deionized water for 3 days and lyophilized to obtain PTX-loaded DA-P-SS-T (DA-P-SS-T/PTX) nanomicelles. The same procedure was used to fabricate PT/PTX and P-SS-T/PTX nanomicelles. Subsequently, a Malvern Zetasizer Nano ZS90 was used to measure the particle size and zeta potential of nanomicelles, and TEM was used to assess the morphology of nanomicelles. HPLC was used to measure drug release from PTX-loaded nanomicelles in vitro (Waters Corp., Waltham, MA, USA). The drug loading capacity (DLC) and drug loading efficiency (DLE) were calculated as described previously [18].

### Cell culture

Human lung adenocarcinoma A549 cells and drug-resistant A549/ADR were from the College of Pharmaceutical Science, Guangzhou Medical University (Guangzhou, China). The cells were cultured in RMPI-1640 (pH 7.4) supplemented with 10% FBS and 1% penicillin/streptomycin at 37 °C in a humidified atmosphere containing 5% CO_2_. For maintenance of drug resistance, A549/ADR cells were cultured in the presence of 4 µM cisplatin (CDDP), and CDDP-free medium was used for 1 week prior to initiation of the experiments [25]. All cell-based protocols were performed according to the guidelines of the Institutional Animal Care Committee and Ethics Committee at Guangzhou Medical University (GZMUC 10-05010).

### Cytotoxicity measurements in vitro

A549 or A549/ADR cells were seeded in a 96-well plate at 1 × 10^4^ cells per well; after 12 h, cell culture medium was replaced with fresh medium containing various nanomicelles. Culture medium alone was used as the blank control. After 48 h, cell viability was determined by proliferation assays [26]. The IC50 values were calculated as drug concentrations that inhibited cell growth by 50% using curve fitting of the cell viability data expressed as the percentage of the control sample data.

### In vitro measurements of apoptosis

A549 and A549/ADR cells were seeded in 6-well plates at 2.5 × 10^5^ cells per well; after 12 h, cell culture medium was replaced with medium containing various nanomicelles. The final concentration of PTX was 10 µM. After 24 h, apoptosis was detected using a FITC Annexin V staining kit and flow cytometry (FACScan) according to the standard protocol [27].

### Cellular uptake

A549 cells were seeded in 6-well plates (5 × 10^4^ cells per well) for 24 h and then incubated with 1 mM 5-(N-ethyl-N-isopropyl)-amiloride, 10 µg/mL chlorpromazine, and 200 µM genistein for 30 min. DA-P-SS-T/C6 (10 µg/mL) nanomicelles were added and incubated at 37 °C for 2 h. Then, the nuclei of the cells were labeled with 2 µg/mL DAPI for 10 min, and the images were acquired by confocal laser scanning microscopy (CLSM) (Zeiss LSM 710).

### Lysosomal escape

A549 or A549/ADR cells (5 × 10^4^) were seeded in a special confocal microscopy dish (NEST) for 24 h and incubated with free C6 or C6-loaded nanomicelles for 4 h. The cells were stained with 1 µM LysoTracker Red for 30 min and 2 µg/mL DAPI for 10 min at 37 °C in the dark, washed with cold PBS, and imaged by CLSM.

### Mitochondrial localization

A549 and A549/ADR cells were inoculated into confocal microscopy dishes. After 12 h, culture medium was replaced with medium containing free C6 or C6-loaded nanomicelles. After 12 h, the solution was removed, and the cells were washed three times with PBS; the cells were treated with a mitochondrial fluorescence probe, MitoTracker Red, for 30 min and imaged directly by CLSM.

### Mitochondrial membrane potential assay and ATP level measurement

Mitochondrial membrane potential was assayed by using a TMRE mitochondrial membrane potential assay kit (Abcam, USA) [28]. Briefly, A549 or A549/ADR cells were seeded in a 96-well plate (1.0 × 10^4^ cells per well) for 24 h and incubated with 5 µg/mL free C6 or C6-loaded nanomicelles. The cells were incubated in the presence of 200 nM TMRE at 37 °C for 20 min. The medium was removed, and the cells were washed three times with PBS and imaged by CLSM.

Additionally, A549/ADR cells were inoculated into 24-well plates for 24 h and then treated with various nanomicelles for 4 h. The intracellular ATP level of the cells was detected by an ATP luminescence assay kit according to the manufacturer’s protocol. The luminescent signal of ATP was assayed by a Lumat LB 9507 luminometer, and the ATP level was calculated using a calibration curve.

### Western blotting and quantitative RT-PCR

A549 and A549/ADR cells were cultured in a 6-well plate at 4 × 10^5^ cells/well at 5 % CO_2_ at 37 °C for 48 h and treated with taxol and PTX-loaded nanomicelles at a final concentration of 10 µM for another 12 h. Western blotting was used to determine the protein levels in human lung cancer cells as described previously [29]. Antibodies against P-gp and β-actin were from Cell Signaling Technology (Beverly, MA, USA). The membranes were rinsed, and the signal was visualized using an enhanced chemiluminescence detection kit.

The expression of P-gp-related genes was analyzed by real-time PCR [30]. In brief, RNA was extracted using a TRIzol® Plus RNA purification kit (Invitrogen). cDNA template was reversed transcribed from the extracted and purified RNA samples (500 ng) using a SuperScript™ III first-strand synthesis supermix (Invitrogen), and standard PCR analysis was performed using Power SYBR® Green PCR master mix (Invitrogen). A real-time PCR detection system (CFX384, Bio-Rad, USA) was used to detect the PCR products after various treatments of A549/ADR cells.

### Inhibition of cell migration in vitro

A549 or A549/ADR cells were seeded in the upper chamber of 24-well Transwell plates (8 μm pores) in medium without FBS (10^5^ cells per well, 100 µL) and cultured with various nanomicelles for 12 h; then, the medium was replaced with medium supplemented with 10% FBS, and the cells were cultured for 24 h. Culture medium and cells that have not invaded the gel were removed from the Transwell chamber, and migrated cells on the bottom surface of the membrane were fixed with 100% methanol for 10 min and stained with 0.1% crystal violet for 30 min for imaging and calculation.

### Pharmacokinetics

Pharmacokinetics of the nanoparticles was studied in ICR mice (Guangdong Medical Animal Experiment Central, Guangzhou, China). Taxol, P-SS-T/PTX, and DA-P-SS-T/PTX were administered via the tail vein at a dose of 10 mg/kg PTX. Blood was sampled at regular intervals from the posterior orbital plexus of mice. Plasma was prepared by centrifugation of the blood samples at 5000 g for 15 min. The content of PTX was determined by HPLC.

### Antitumor studies and toxicity analysis

All animal experiments were conducted according to the guidelines of the Institutional Animal Care and Use Committee of the Animal Experiment Center of Guangzhou Medical University (Guangzhou, China) and the Regulations for the Administration of Affairs Concerning Experimental Animals. A549/ADR cells were subcutaneously injected into the right flank of mice. Tumor growth and body weight were recorded every other day. When the tumor volume reached approximately 100 mm^3^, the animals were randomly divided into five groups: PBS, Taxol (15 mg/kg), PT/PTX (15 mg/kg), P-SS-T/PTX (15 mg/kg), and DA-P-SS-T/PTX (15 mg/kg) (n = 5). Seven days after the first injection via the tail vein, mice were again administered with 100 µL of the same nanoparticles. After 24 days of the treatment, tumor-bearing nude mice were sacrificed, and the tumors in each group were imaged and weighed. The relative tumor volume was calculated as the length × width^2^/2 (mm^3^). The tumor volume inhibition was calculated as reported previously [31]. One day after the last injection, the tumor tissues were harvested for ATP and P-gp assays, and the kidney, spleen, and liver were fixed in 4% formaldehyde for H&E staining. The blood of the mice was collected for blood routine examination.

### Statistical analysis

Statistical analyses were performed using GraphPad Prism 5.0 software. The data are expressed as the mean ± SD. One-way ANOVA was used to determine significance of the differences using the following annotations: n.s., *P < 0.05, **P < 0.01, and ***P < 0.001.

## Results and discussion

### Preparation and characterization of DA-P-SS-/PTX nanomicelles

**Scheme 2:**
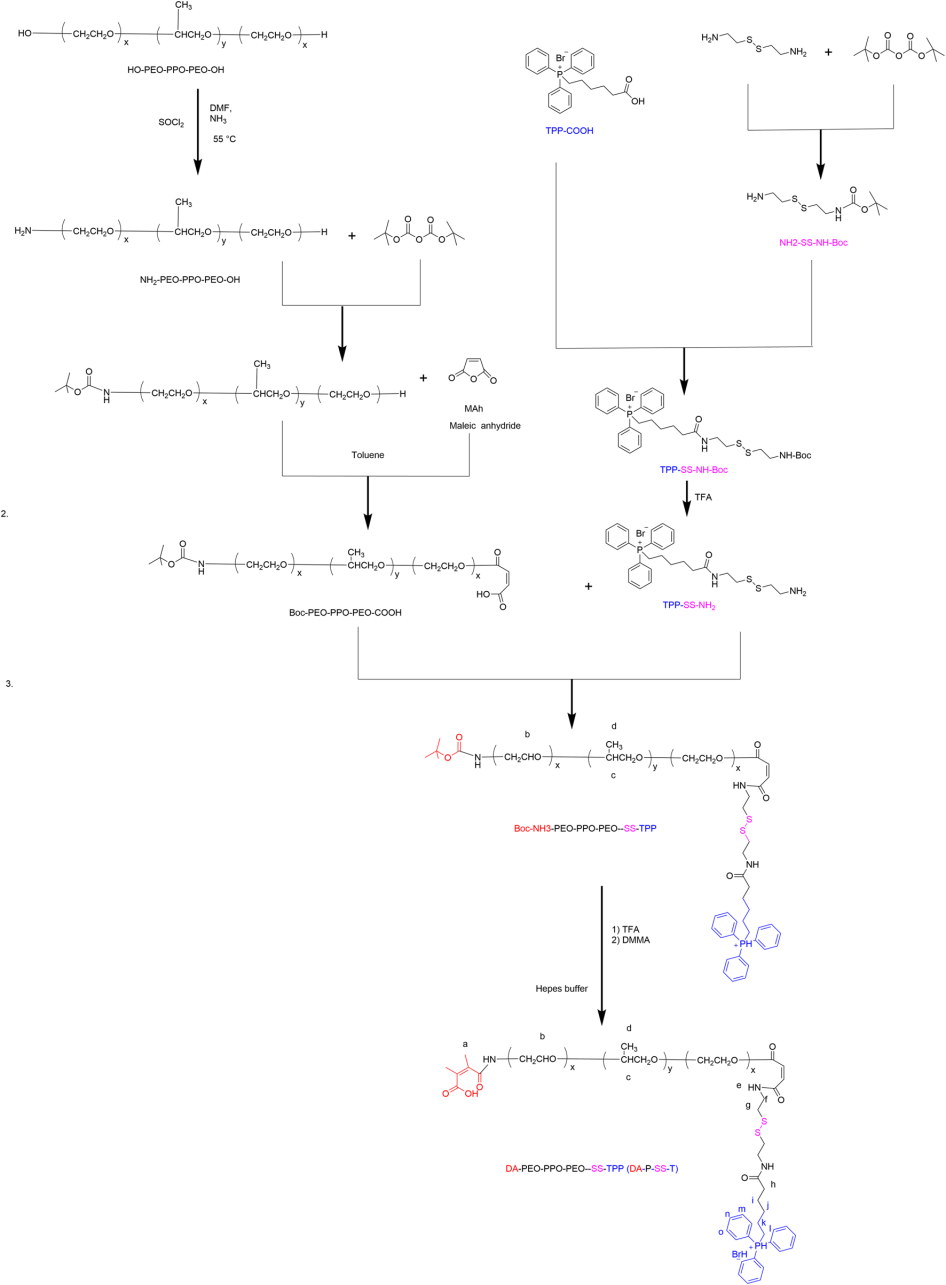
Scheme of the synthesis of DA-P-SS-T

**Fig. 1:**
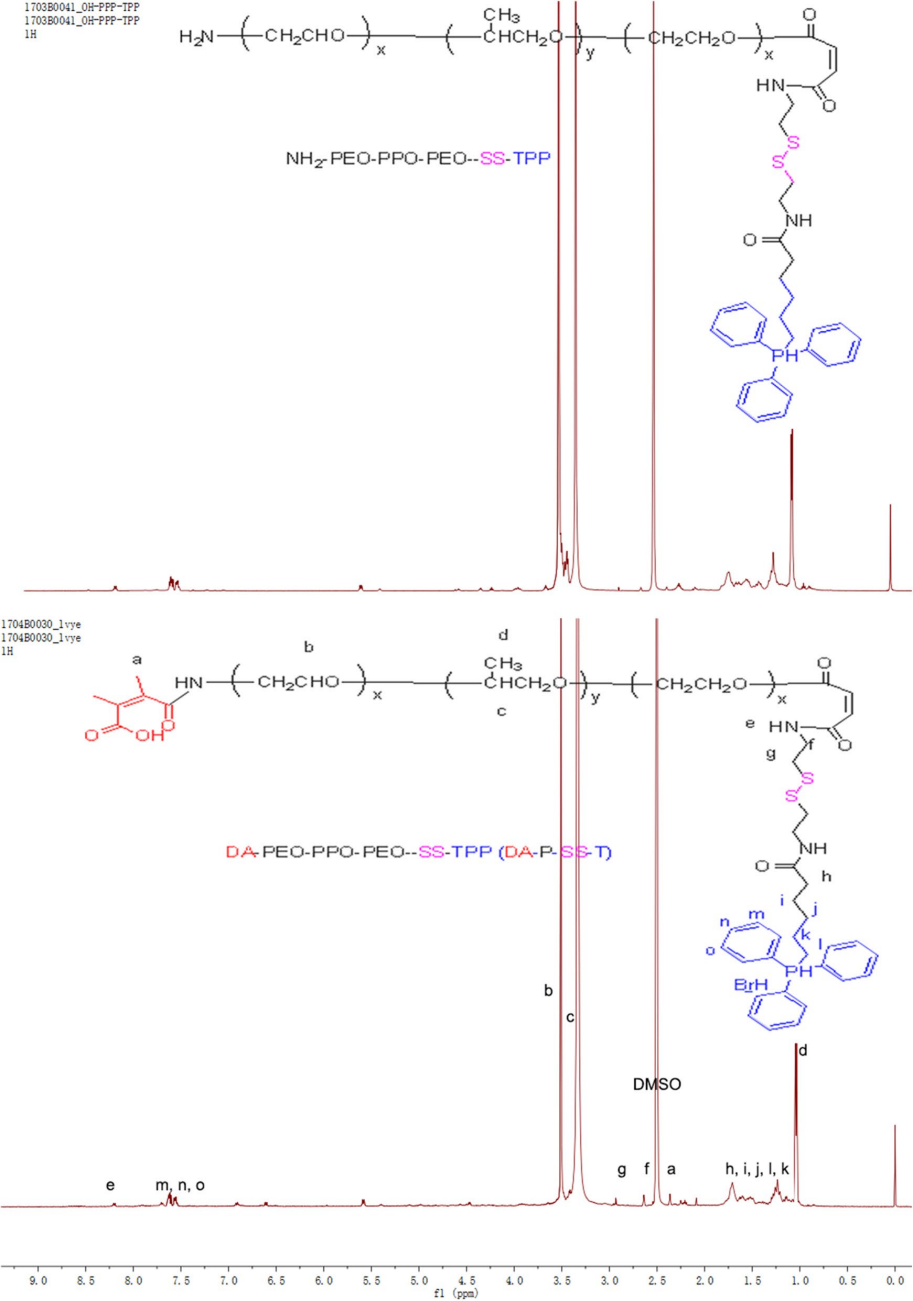
^1^ H NMR spectra of P-SS-T and DA-P-SS-T polymers

DA-P-SS-/P was synthesized according to Scheme 2. The whole process was divided into two parts. Initially, P85 was conjugated to COOH-TPP to produce OH-P85-SS-TPP (P-SS-T) (Fig. 1). DA-P-SS-T was obtained by DA modification of the amino groups of NH_2_-P85-SS-TPP to extend circulation in the blood. In addition to the aromatic ring of TPP (8.06–7.65 ppm) and the methylene group of P85 (3.32 ppm), the ^1^ H-NMR resonance peak at 2.43 ppm was assigned to the methyl groups of the DA residue, indicating successful synthesis of DA-P-SS-T. Dynamic light scattering (DLS) indicated that the average diameter of DA-P-SS-T/PTX nanomicelles was approximately 160 nm, which was approximately 5 nm larger than that of P-SS-T/PTX nanomicelles (Table 1). The average diameters of DA-P-SS-T/PTX and P-SS-T/PTX nanomicelles were not significantly different; however, the charge of DA-P-SS-T/PTX with a negative potential of −8.65 mV was significant less than that of P-SS-T/PTX, indicating successful modification of P-SS-T by DA.

**Table 1:** Characterization of nanomicelles

Foumulation	Particle size (nm)	PDI	Zeta potential (mV)	DLC (%)	DLE (%)
PT/PTX	145	0.215	7.45	5.12	82.55
P-SS-T/PTX	155	0.193	7.82	5.82	77.54
DA-P-SS-T/PTX	160	0.231	−8.65	6.01	72.32
PT	130	0.152	7.42		
P-SS-T	140	0.186	7.65		
DA-P-SS-T	145	0.168	−10.05		

**Fig. 2:**
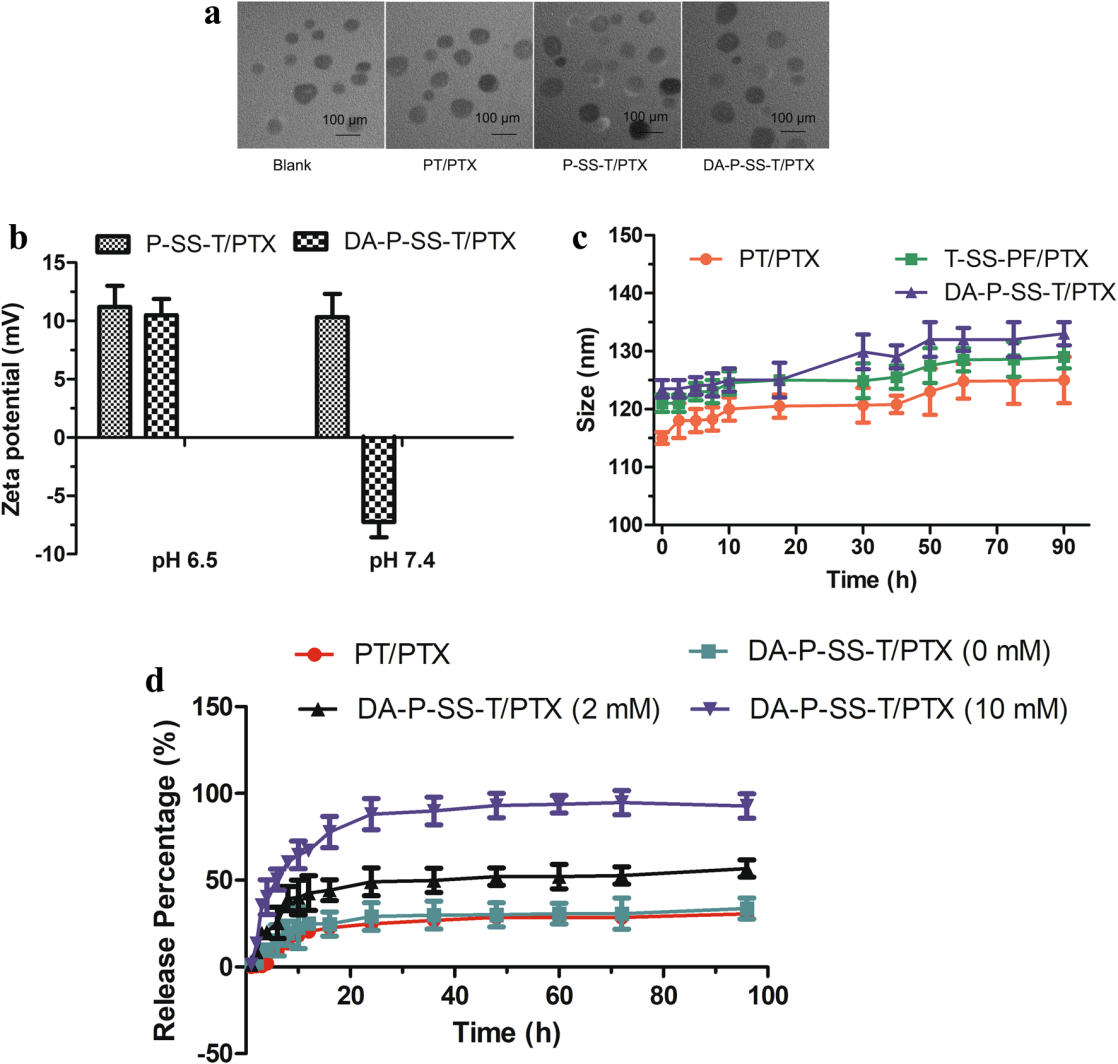
Characterization of DA-P-SS-T/PTX nanomicelles and stimulus-responsive release of PTX. **A** TEM images of nanomicelles. **B** The changes in the zeta-potential of P-SS-T/PTX and DA-P-SS-T/PTX nanomicelles during incubation in PBS at pH 6.5 and 7.4. **C** The changes in the size of nanomicelles during incubation in PBS containing 10% FBS at 37 °C for 90 h. **D** In vitro PTX release from DA-P-SS-T/PTX nanomicelles in PBS in the presence of various GSH concentrations

The amphipathic DA-P-SS-T and PTX formed spherical micelles by self-assembly in aqueous solution (Fig. 2A). The size of the nanoparticles detected in TEM images was smaller than that estimated by DLS. DLS was used to evaluate the size of hydrated nanoparticles. However, nanoparticles detected by TEM were in a dry state. Therefore, the differences in the nanoparticle size evaluated by DLS and TEM may be due to different states of the nanoparticles.

The pH-sensitive reversal of the charge of DA-P-SS-T/PTX nanomicelles was investigated over time to assess the stability of DA-P-SS-T/PTX nanomicelles. The changes in the surface charge of P-SS-T/PTX and DA-P-SS-T/PTX were measured in PBS at various pH (Fig. 2B). The zeta potential of DA-P-SS-T/PTX nanomicelles was significantly increased at pH 6.5, and DA-P-SS-T/PTX nanomicelles remained negatively charged at pH 7.4; furthermore, the zeta potential of these nanomicelles was increased only slightly over 24 h. In contrast, P-SS-T/PTX nanomicelles maintained a positive surface charge at pH 6.5 and 7.4 due to high positive surface charge of TPP [32]. Low pH-induced hydrolysis of DA-conjugated amides was responsible for the conversion of the carboxylic groups into the amine groups, resulting in the recovery of the mitochondria-targeting ligand of DA-P-SS-T/PTX nanomicelles. Hence, charge reversal triggered by the tumor microenvironment may provide for extended circulation time and simultaneous effective mitochondrial targeting [33]. Moreover, particle size was not significantly changed after incubation in medium containing 10 % FBS for 96 h, indicating excellent stability of the three nanomicelle constructs (Fig. 2C).

As shown in Fig. 2D, the release of PTX from all formulations was characterized by an initial burst. In the absence of GSH, the cumulative release of PTX from DA-P-SS-T/PTX nanomicelles reached 25% after 15 h. Subsequent release rate was notably slower, and only 30.5% of PTX was released from DA-P-SS-T/PTX nanomicelles after 96 h. In contrast, the assay of DA-P-SS-T/PTX nanomicelles in the presence of 2 mM or 10 mM GSH indicated a considerable increase in the cumulative release of PTX to approximately 46.2 and 85.7%, respectively, after 96 h. These results suggested that DA-P-SS-T/PTX nanomicelles rapidly released the drug in a reducing environment and did not release PTX in a nonreducing environment. The results showed that the structure of the micelles suppressed the leakage of the therapeutic agent into the circulation and allowed rapid drug release in the tumor cells.

### Cellular uptake

**Fig. 3:**
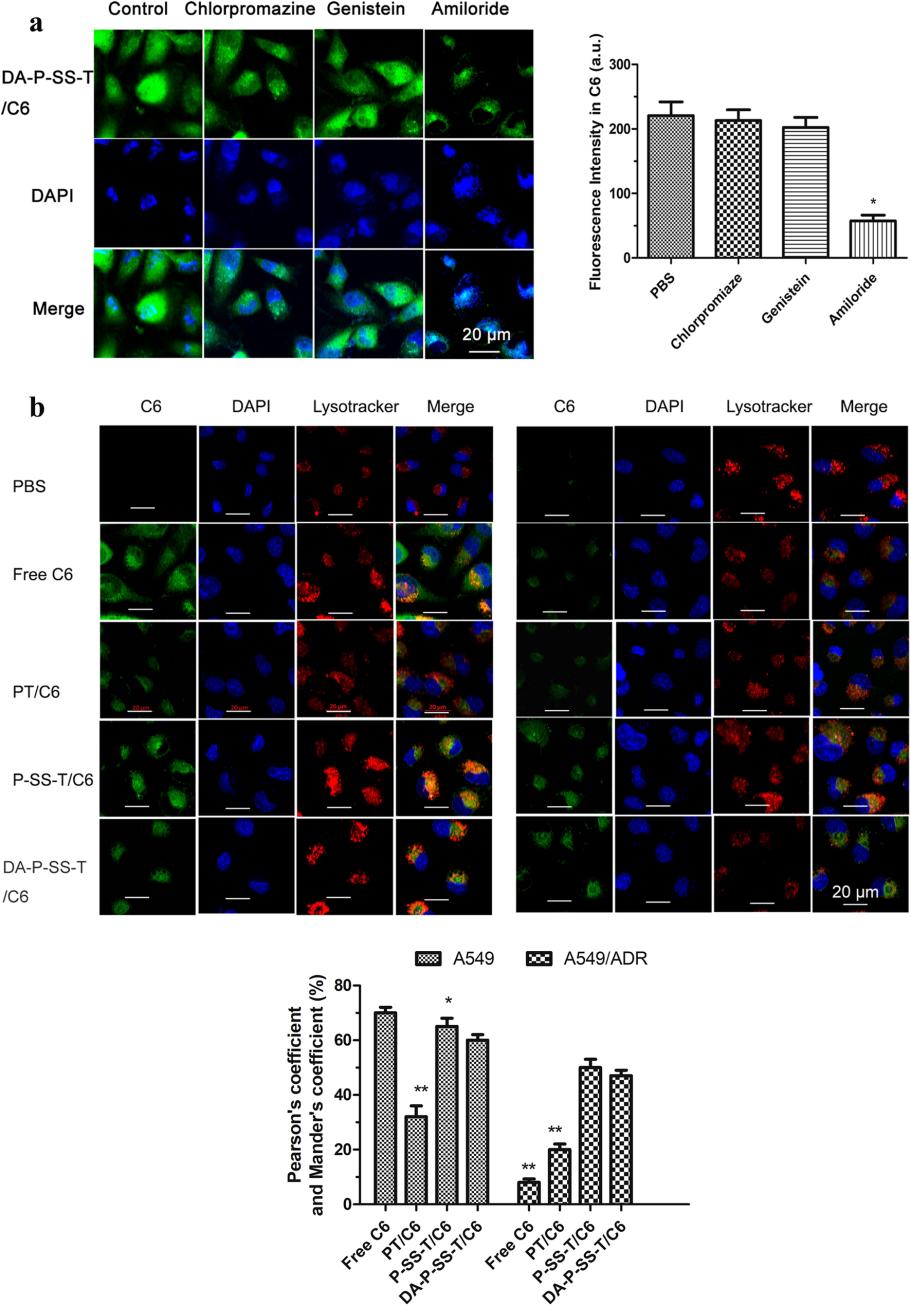
Cellular uptake and lysosomal escape of DA-P-SS-T/PTX nanomicelles. **A** CLSM images of A549 cells pretreated with chlorpromazine, genistein, and amiloride and then treated with DA-P-SS-T/C6 nanomicelles. The nuclei were stained by DAPI (blue). *P < 0.05, vs. PBS. **B** CLSM images of lysosomal escape of nanomicelles in A549 and A549/ADR cells. Green fluorescence signal corresponds to C6. The late endosomes and lysosomes were labeled by LysoTracker Red. Yellow fluorescence signal corresponds to overlay of the C6 and lysosomal signals. Scale bars are 20 μm. (1) CLSM; (2) The intensity of the fluorescence signal in the corresponding images or Pearson coefficient and Mander coefficient. *P < 0.05, **P < 0.01 vs. DA-P-SS-T/PTX

The loss of DA from DA-P-SS-T/PTX nanomicelles in the tumor tissue results in the absorption of positively charged P-SS-T/PTX nanomicelles on tumor cells due to electrostatic interaction and subsequent entry through endocytosis, similar to TPH/PTX [18]. Macropinocytosis-mediated endocytosis was the main endocytic pathway for DA-P-SS-T/coumarin-6 (C6) nanomicelles (Fig. 3A). The carriers are initially trapped in the lysosomes; thus, effective drug carriers have to successfully and rapidly escape the lysosome. Therefore, the interaction between the lysosomes and nanoparticles was studied by CLSM. Pearson coefficient- and Mander coefficient-based quantification of the colocalization volume [34] demonstrated that the order of the mean intensity of the fluorescence signal in A549/ADR cancer cells was PBS < free C6 < PT/C6 nanomicelles < DA-P-SS-T/C6 nanomicelles < P-SS-T/C6 nanomicelles, indicating the influence of DA due to charge shielding of DA-P-SS-T/C6 nanomicelles (47.6%) versus DA-lacking P-SS-T/C6 nanomicelles (50.4%) (Fig. 3B). This result demonstrated that P-SS-T/PTX is slightly superior to DA-P-SS-T/PTX in the process of cell entry and targeting of the mitochondria to induce apoptosis due to advantageous positive charge.

### Significance of mitochondrial targeting

**Fig. 4:**
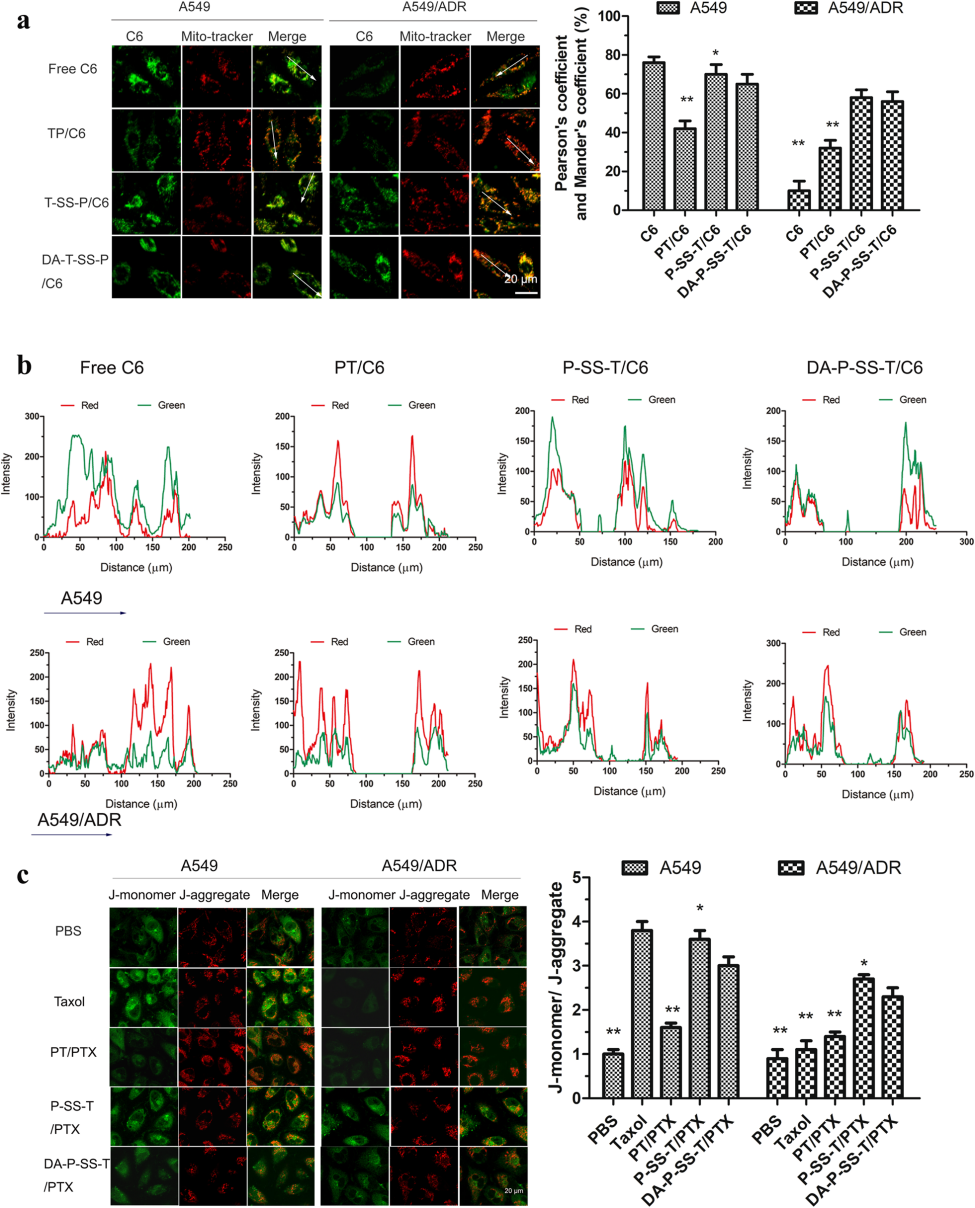
In vitro mitochondria-targeting delivery and assay of the mitochondrial membrane potential. **A** Tracking of C6 delivery in A549 and A549/ADR cells based on the MitoTracker signal corresponding to the mitochondria (red) and C6 signal (green) by CLSM. *P < 0.05, **P < 0.01 vs. DA-P-SS-T/C6. **B** Colocalization analysis. **C** A549 and A549/ADR cells treated with DA-P-SS-T/C6 were labeled with JC-1 to detect mitochondrial depolarization by CLSM and compare the signal with that in the control cells. Green and red colors correspond to the signal of monomeric and aggregated forms of JC-1, respectively. (1) CLSM image; (2) Pearson coefficient and Mander coefficient or the JC-1 monomer/JC-1 aggregate ratio

After escaping the lysosomes, DA-P-SS-T/C6 nanomicelles were expected to be localized and transported to the mitochondria due to the effect of TPP. This process was evaluated by CLSM with colocalization analysis in A549 and A549/ADR cells. The treatments included nanomicelles lacking redox responsiveness (free C6 and PT/C6) and nanomicelles with redox responsiveness (P-SS-T/C6 and DA-P-SS-T/C6). The mitochondria of A549 cells were specifically labeled with MitoTracker Red, and green fluorescence signal of C6 was detected by CLSM (Fig. 4A). Superposition of green and red produced yellow color in the images indicating that the C6 signal overlaps with the mitochondria. Clear separation of the green and red signals indicated that C6 was located in other subcellular organelles, for example, in the microtubules, suggesting further that taxol colocalized with the microtubules and was not located in the mitochondria. A high GSH level in the cytoplasm results in the cleavage of the disulfide bonds in the micelle matrix, resulting in the rapid release of encapsulated C6 by the micelles [35]. C6 released in the cytoplasm diffused into the microtubules. In the case of the treatment with TPP-functionalized micelles (PT/C6, P-SS-T/C6, and DA-P-SS-T/C6), the mitochondria in the merged image were yellow, indicating a good overlap between the green signal of C6 and the red signal of the mitochondria. Furthermore, only a few green fluorescence spots were detected in other subcellular organelles. Figure 4B shows the red and green signals of the mitochondria and C6, respectively, similar to Fig. 4A. The intensity of the fluorescence signal of free C6 was randomly distributed in the images. However, the signals of TPP-functionalized micelles were characterized by almost synchronous distribution of the green and red spots despite variable intensity of the fluorescence signals of various types of micelles. Interestingly, a few green spots in the P-SS-T/C6 and DA-P-SS-T/C6 groups were located outside of the mitochondria. Comparison with the green signal in the Taxol group indicated high intensity of green fluorescence in A549/ADR cells in the P-SS-T/C6 and DA-P-SS-T/C6 groups, suggesting that P-SS-T/C6 and DA-P-SS-T/C6 were able to overcome drug resistance. These results demonstrated that the DA layer was removed from the micelles to expose TPP, which provided for efficient mitochondrial targeting. These processes resulted in specific accumulation of the nanomicelles in the mitochondrial fraction, contributing to enhanced membrane permeability of the mitochondria and ultimately leading to direct diffusion of released PTX into the mitochondria.

The green and red signals did not completely overlap in the mitochondrial region, and the intensity of the green signal was higher than that of the red signal, indicating that DA-P-SS-T/C6 entered the mitochondria more rapidly than other agents. In particular, the differences in the rate of the mitochondrial entry in A549 cells and A549/ADR cells were significant. Pearson coefficient- and Mander coefficient-based quantification of the volume of colocalization corresponded to 54.2% of overlapping regions for DA-P-SS-T/C6 in A549/ADR cells, indicating higher intensity of the yellow signals obtained in the case of these nanomicelles compared with the signals observed in the case of PT/C6 and suggesting that the treatment with DA-P-SS-T/C6 resulted in substantial accumulation of nanomicelles in the mitochondria.

Demonstration of trafficking into the mitochondria and successful release of the drugs was followed by the detection of the effect of DA-P-SS-T/PTX nanomicelles on the mitochondrial membrane potential using the fluorescent probe 5,5’,6,6’-tetrachloro-1,1’,3,3’-tetraethylbenzimidazolcarbocyanine iodide (JC-1). The data of Fig. 4C showed strong green fluorescence with barely detectable red fluorescence in the cells treated with DA-P-SS-T/C6 nanomicelles, clearly indicating a dramatic decrease in the mitochondrial membrane potential and the destruction of membrane integrity. In contrast, both green and red fluorescence signals were detected in other treatment groups. Thus, TPP-functionalized micelles were able to reduce the mitochondrial membrane potential [36].

**Fig. 5:**
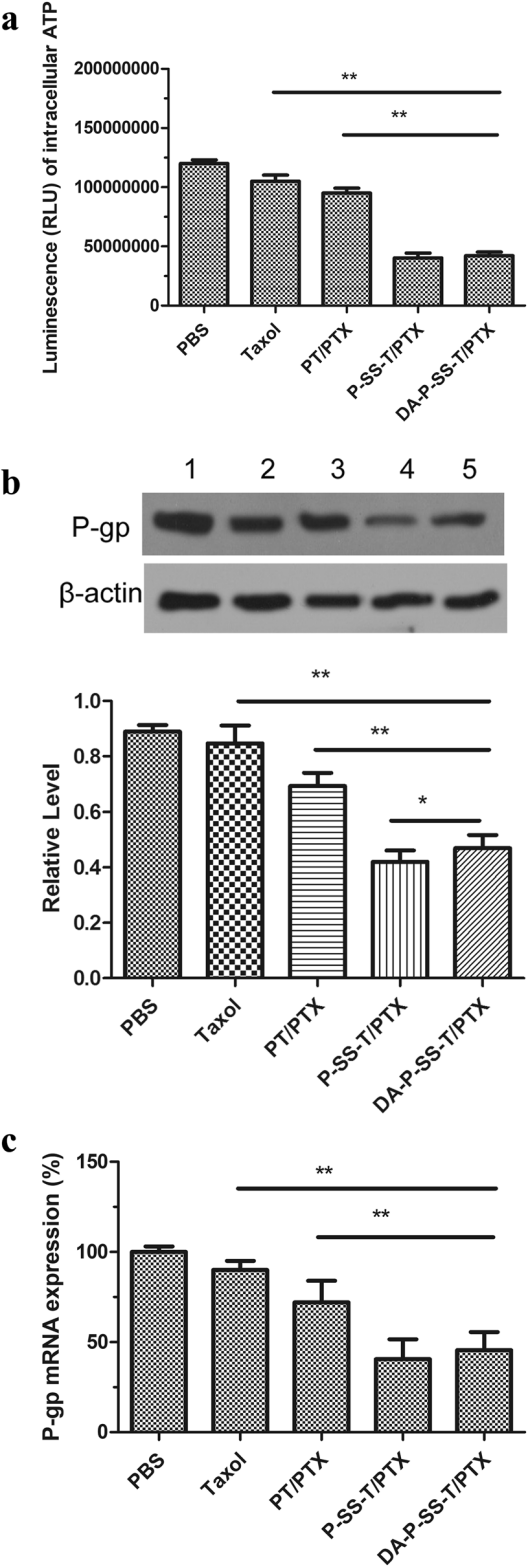
Reversal of drug resistance by DA-P-SS-T/PTX nanomicelles. Intracellular ATP levels (**A**), the levels of P-gp protein (**B**), and relative expression of P-gp mRNA (**C**) in A549/ADR cells treated with various agents. (1) Western blot; (2) quantitative analysis of relative expression of P-gp. *P < 0.05, **P < 0.01

ATP from the mitochondria contributes to the overexpression of drug efflux pumps to induce MDR, suggesting that damage of the mitochondria and a reduction in ATP levels can effectively reverse drug resistance [37]. As shown in Fig. 5A, Taxol had a weak effect on the ATP levels in A549/ADR cells because the drug was pumped out of the tumor cells. However, nanomicelles with TPP functionalization induced a stepwise decrease in the intracellular ATP levels, and DA-P-SS-T/PTX nanomicelles were even stronger suppressors of ATP levels than PT/PTX nanomicelles. The differences between these two groups may be attributed to mitochondrial targeting of PTX release from DA-P-SS-T/PTX nanomicelles in the context of intracellular GSH. Thus, a polymer with TPP can be used as a potent vector for targeted delivery of PTX to the mitochondria essential for maximized destructive effects of PTX confirmed by alterations in the mitochondrial membrane potential. ATP downregulation may be associated with a decrease in the expression of P-gp. PTX induced a decrease in the mitochondrial membrane potential followed by a reduction in the ATP level that blocked the energy supply and significantly suppressed biological activity of P-gp to eventually overcome drug resistance of A549/ADR cells.

To confirm these suggestions, P-gp expression was measured by western blot and RT-PCR in cells treated with DA-P-SS-T/PTX nanomicelles. The data of Fig. 5B indicated that the treatment of A549/ADR cells with PBS, Taxol, or PT/PTX nanomicelles had no significant effect on the expression of P-gp. However, the treatment with P-SS-T/PTX and DA-P-SS-T/PTX nanomicelles induced a considerable decrease in the level of P-gp down to 38.5 and 44.7% of that in the corresponding controls, respectively (Fig. 5B-2). These results were similar to the data of RT-PCR (Fig. 5C). These findings demonstrated the mechanism by which DA-P-SS-T/PTX nanomicelles overcome drug resistance, involving nanomicelle targeting, depletion of mitochondrial membrane potential, and reduction in ATP levels.

### In vitro evaluation of antimetastatic activity

**Fig. 6:**
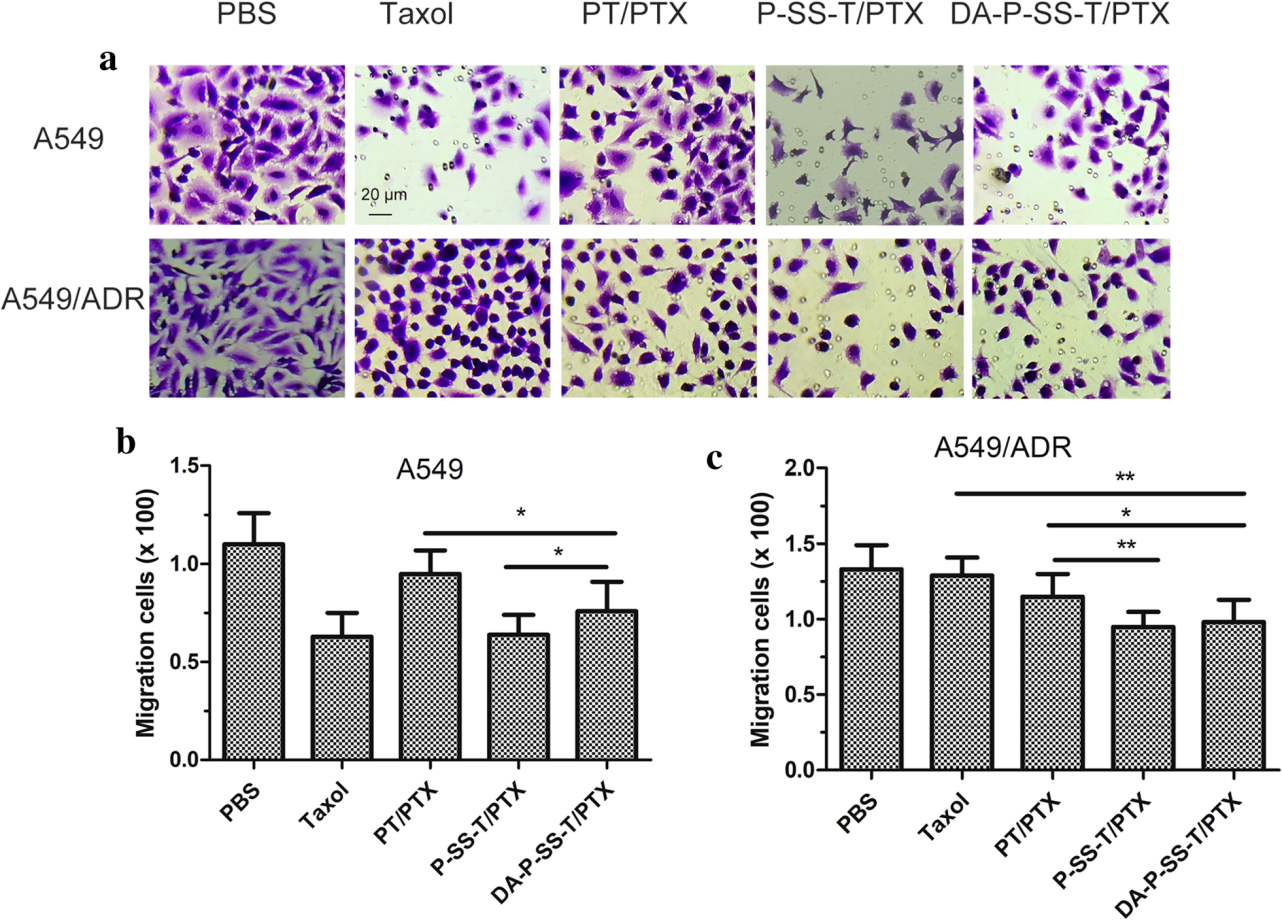
The migration of A549 and A549/ADR cells. The representative images (**A**) and quantification of migrating A549 cells (**B**) and A549/ADR cells (**C**) (n = 4). Scale bar, 20 μm. *P < 0.05; **P < 0.01

Metastasis to distant sites is the key pathological process leading to cancer relapse even after resection of primary tumors [38]. ATP indirectly or directly induces metastasis via tumor-derived microvesicles [39]. As shown in Fig. 6A, P-SS-T/PTX and DA-P-SS-T/PTX nanomicelles inhibited the migration of A549 and A549/ADR cells, indicating an excellent suppression of lateral migration. P-SS-T/PTX treatment resulted in a lower number of migrating cells compared with that detected after DA-P-SS-T/PTX treatment; however, the inhibitory effects of these treatments on the migration of A549/ADR cells were not significantly different (Fig. 6B, C), suggesting that the inhibitory effect of DA-modified P-SS-T/PTX on cell migration is more potent. Thus, DA-P-SS-T/PTX nanomicelles significantly suppressed the lateral migration by reducing the ATP level and blocking the energy supply.

### Assay of cytotoxicity and apoptosis in vitro

**Fig. 7:**
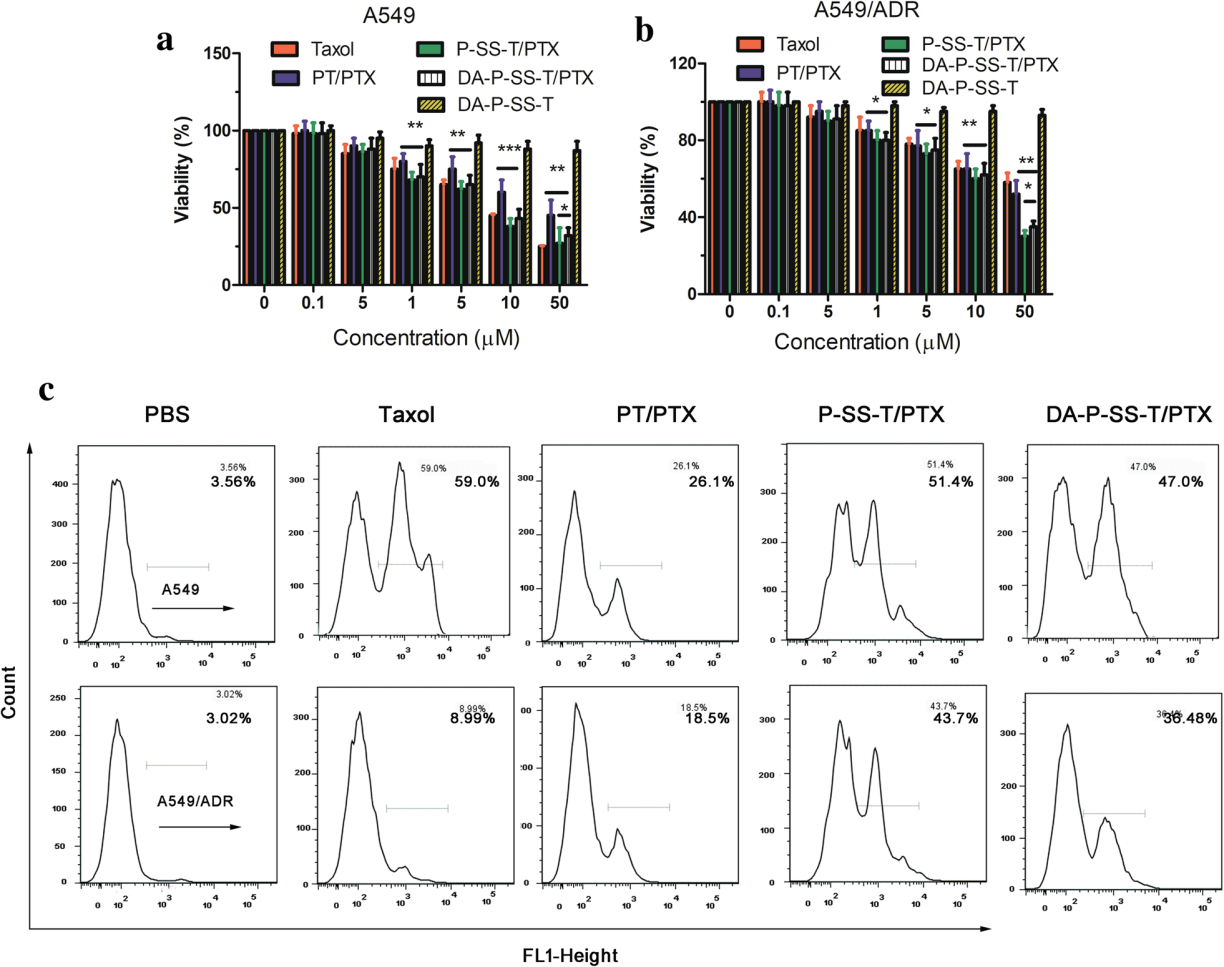
Antiproliferative ability in vitro. Cytotoxicity of PTX-loaded nanomicelles against A549 (**A**) and A549/ADR (**B**) cells after 48 h. **C** The apoptosis rate detected by flow cytometry. The data are presented as the mean ± standard deviation (n = 3). *P < 0.05; **P < 0.01

Subcellular localization of DA-P-SS-T/PTX nanomicelles provided conditions aggravating mitochondrial destruction. Inhibition of cancer drug resistance by DA-P-SS-T/PTX nanomicelles was investigated in vitro by proliferation assays. The data of Fig. 3 indicated that the viability of cancer cells was decreased concomitant to an increase in PTX concentration. Treatment with Taxol and PTX-loaded nanomicelles resulted in concentration-dependent cytotoxicity in A549 cells (Fig. 7A). However, the survival rate of A549/ADR cells was only slightly reduced after incubation with Taxol and PT/PTX nanomicelles even at high concentrations, indicating that A549/ADR cells were relatively resistant to PTX (Fig. 7B). However, extended treatment with P-SS-T/PTX and DA-P-SS-T/PTX resulted in high antitumor activity against A549/ADR cancer cells. The IC50 for DA-P-SS-T/PTX (19.45 µM) was lower than that for Taxol (89.49 µM) and PT/PTX nanomicelles (60.99 µM) (Table 2) apparently due to the rapid release of PTX in the presence of high concentration of GSH in the cells and mitochondrial targeting. Moreover, the cytotoxicity of DA-P-SS-T in lung cancer cells was low, indicating that these carriers are safe and biocompatible with tissues and cells.

**Table 2:** The IC50 values in A549 and A549/PTX cells incubated in the presence of Taxol and nanomicelles for 48 h (n = 5)

Cells\ Drugs	Taxol	PT/PTX	P-SS-T/PTX	DA-P-SS-P/PTX
A549	8.04	27.32	8.20	9.81
A549/ADR	89.49	60.99	16.32	19.45

The antiproliferative ability of DA-P-SS-T/PTX was also assessed by detecting apoptosis by flow cytometry. As shown in Fig. 7C, the apoptosis rates were 3.56, 59.0, 26.1, 51.4, and 47.0% in A549/ADR cells and 3.02, 8.99, 18.5, 43.7, and 36.48% in A549 cells treated with PBS, Taxol, PT/PTX, P-SS-T/PTX nanomicelles, and DA-P-SS-T/PTX nanomicelles, respectively, indicating that DA-P-SS-T/PTX nanomicelles were able to effectively overcome drug resistance.

### Pharmacokinetics

**Fig. 8:**
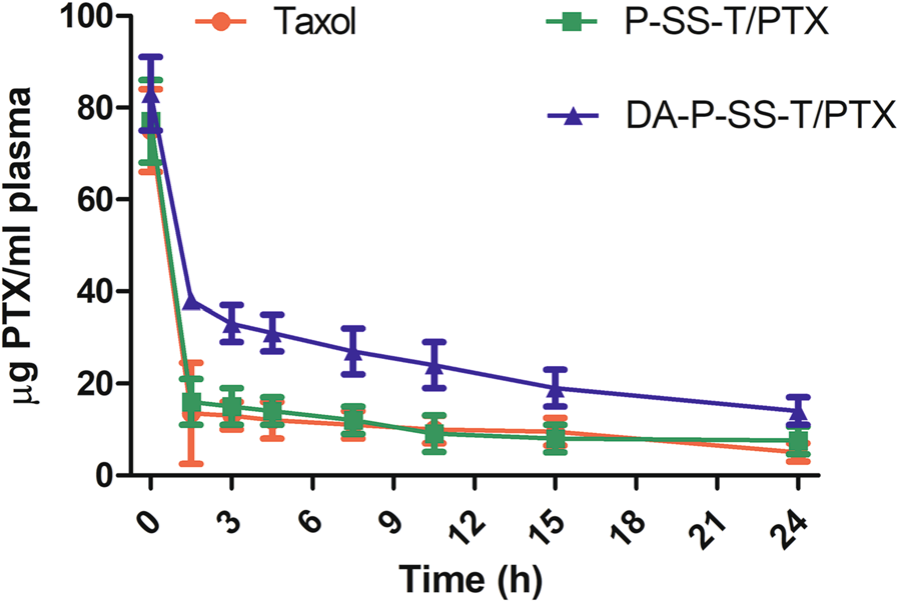
Plasma PTX concentration versus time after intravenous administration of Taxol, P-SS-T/PTX, and DA-P-SS-T/PTX nanomicelles

Pharmacokinetics of Taxol, PT/PTX, P-SS-T/PTX, and DA-P-SS-T/PTX nanomicelles were evaluated in ICR mice after a single intravenous injection to determine the effect of DA modification of the nanoparticles on circulation time of the nanomicelles in the blood. The data of Fig. 8 indicated that negatively charged DA-P-SS-T/PTX nanomicelles had a longer circulation half-life and considerably larger area under the curve than those of electropositive Taxol, PT/PTX nanomicelles, and P-SS-T/PTX nanomicelles. These results indicated the beneficial effect of negatively charged nanoparticles on the extension of circulation time in the blood, which may facilitate nanoparticle accumulation at the tumor sites.

### Evaluation of anticancer efficacy and safety in drug-resistant human lung cancer xenografts

**Fig. 9:**
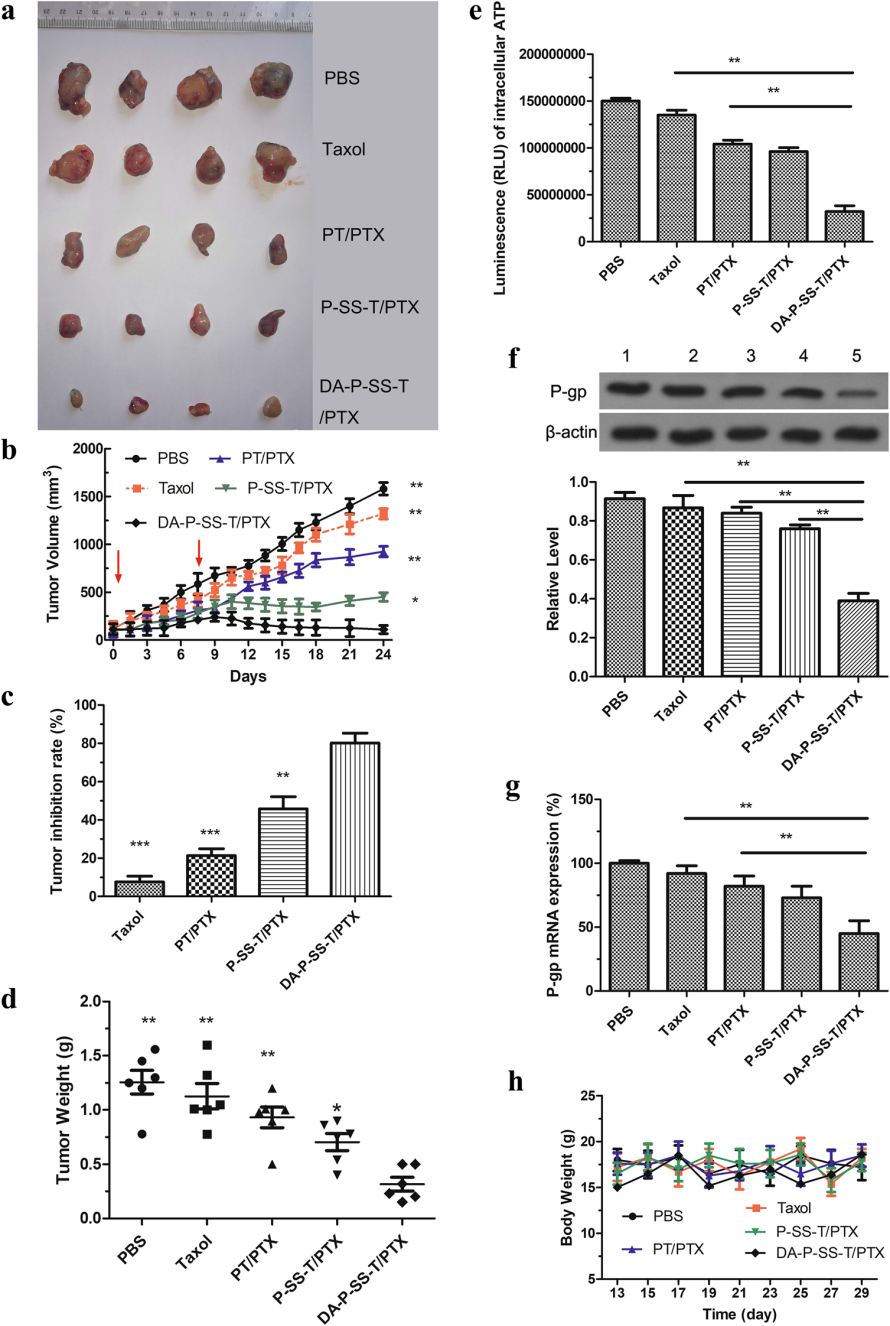

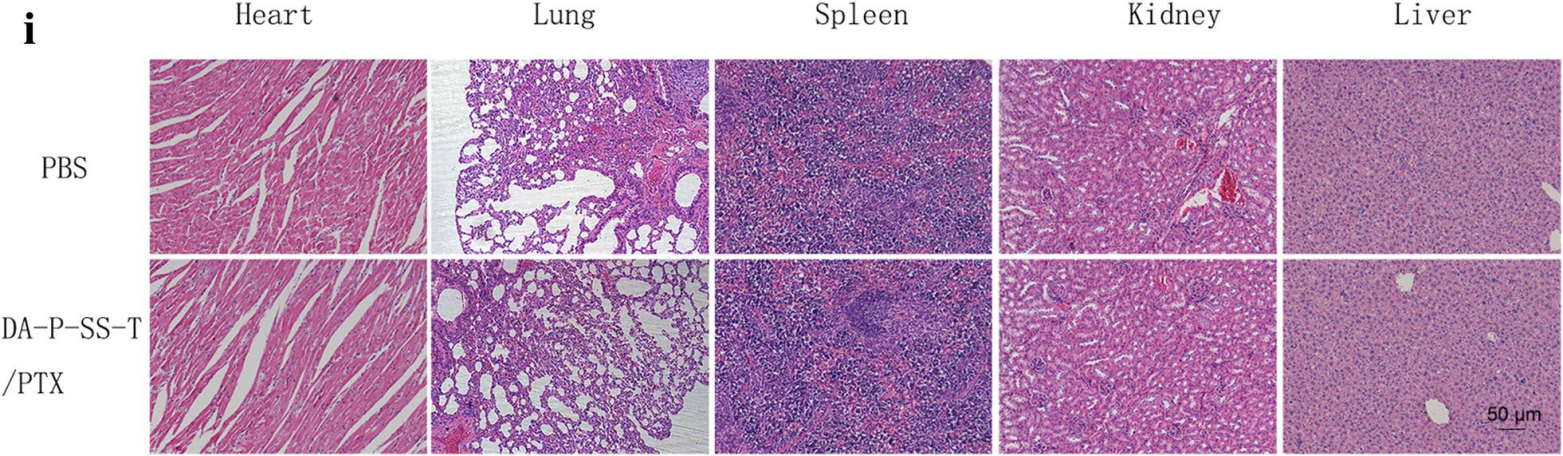
Antitumor and preliminary safety studies of DA-P-SS-T/PTX nanomicelles in resistant cancer xenografts. The representative tumor tissues (**A**) and tumor growth curves (**B**) after intravenous injection of Taxol and self-assembled nanomicelles (red arrows indicate the days of injection). (C) TIR and the corresponding average tumor weight (**D**) after various treatments. TIR was calculated using the following equation: TIR (%) = [1 − X/Y] × 100%, where X corresponds to the mean weight of the tumors in the experimental group and Y corresponds to the mean weight of the tumors in the control groups. Intracellular ATP levels (**E**), western blot assay of the levels of P-gp (**F**), and quantitative analysis of relative expression of P-gp (**G**) in tumor tissue after various treatments. Body weight (**H**) and H&E (**I**) staining of the organ section of A549/ADR xenograft-bearing nude mice treated with various formulations in vivo. (1) Western blot; (2) quantitative analysis of relative expression of P-gp. The data are presented as the mean ± standard deviation (n = 5); *P < 0.05, **P < 0.01, ***P < 0.001 compared with the DA-P-SS-T/PTX nanomicelles

To establish the clinical and translational potential of PTX-loaded nanoparticles, various formulations containing the same dose of PTX were analyzed in therapeutic studies using a xenograft of resistant A549/ADR cells in nude mice. As shown in Fig. 9 A and B, DA-P-SS-T/PTX nanomicelles demonstrated the strongest antitumor activity and substantially inhibited tumor growth. The highest tumor inhibition rate of 81.52% was achieved on day 24 in mice treated with DA-P-SS-T/PTX nanomicelles, which was 8.09-, 3.87-, or 2.02-fold higher than that achieved using Taxol, PT/PTX nanomicelles, or P-SS-T/PTX nanomicelles (Fig. 9C). This result was confirmed by measurements of the tumor weight in the groups (Fig. 9D), suggesting that DA in DA-P-SS-T/PTX nanomicelles extended circulation in the body and provided for the exposure of positively charged P-SS-T/PTX nanomicelles due to rapid decomposition in an acidic environment to increase the electrostatic absorption and permeability in drug-resistant tumors. This result explains why reversal of drug resistance and inhibition of tumor metastasis in vitro by DA-P-SS-T/PTX nanomicelles is slightly weaker than the effects of P-SS-T/PTX nanomicelles. Additionally, rapid release of PTX due to the reduction of the disulfide bond in the presence of high GSH concentrations enhanced the cytotoxic effect on drug-resistant cancer cells due to an increase in the concentration of PTX. The effects of these nanomicelles on ATP level and P-gp expression supported these considerations (Fig. 9E, F, G).

The data of Fig. 9H indicated that DA-P-SS-T/PTX treatment of mice did not induce significant changes in the body weight compared with that in other groups. In addition, negligible systemic toxicity was also verified by the H&E staining analysis of the sacrificed organs and routine blood examination (Fig. 9I; Table 3). Apparently, DA modification of P-SS-T reduced the degradation of the nanomicelles by the macrophages and influence on other organs and extended the circulation time in the blood thus increasing PTX accumulation in the tumor tissue, enhancing permeability and retention of PTX and providing for mitochondrial targeting due to redox-responsive properties to eventually overcome drug resistance. The use of pluronic-modified materials in DA-P-SS-T/PTX nanomicelles improved the pharmacokinetic profile of PTX and increased the uptake of DA-P-SS-T/PTX nanomicelles.

**Table 3:** Hematological parameters of the mice on the 24st day

	PBS	P-P/ptx	P-SS-P/PTX		DA-P-SS-P/PTX	Reference Range
WBC (10^9^/L)	8.5 ± 4.25	7.25 ± 5.25	7.36 ± 9.25		4.56 ± 0.85	0.8–6.8
Lymph (10^9^/L)	7.85 ± 10.32	6.65 ± 1.35	6.05 ± 2.32		2.65 ± 0.35	0.7–5.7
Mon (%)	2.32 ± 0.05	1.32 ± 0.35	1.32 ± 0.23		0.25 ± 0.07	0.0 –0.3
Gran (10^9^/L)	9.02 ± 3.52	2.32 ± 0.85	1.65 ± 0.35		0.15 ± 0.04	0.1 –1.8
RBC (10^12^/L)	7.35 ± 3.02	7.32 ± 2.56	7.86 ± 3.65		7.69 ± 4.52	6.36–9.42
HGB (g/l)	99.36 ± 56.23	115.6 ± 35.63	125.6 ± 45.3		120.5 ± 52.3	110–143
HCT (%)	32.5 ± 10.35	33.56 ± 10.35	41.25 ± 10.32		36.25 ± 5.65	34.6–44.6
MCV (fl.)	45.26 ± 1.25	41.35 ± 3.25	41.24 ± 1.32		40.56 ± 1.85	48.2–58.3
MCH (pg)	13.2 ± 3.25	16.52 ± 3.25	17.25 ± 2.85		17.56 ± 3.14	15.8–19
MCHC (g/l)	301.1 ± 85.54	322.5 ± 102.5	345.8 ± 112.3		335.6 ± 132.5	302–353
RDW (%)	16.35 ± 3.12	18.52 ± 3.02	15.35 ± 2.32		16.35 ± 4.56	13–17
MPV (fl.)	6.23 ± 0.23	6.10 ± 0.15	5.85 ± 0.65		5.45 ± 0.85	3.8 –6.0

## Conclusions

The present study investigated pH- and redox-responsive mitochondria-targeting nanoparticles. DA contributed to the negatively charged surface of the nanoparticles, which resulted in extended circulation time in the blood. The acidic environment of the lung cancer tissue promoted the shedding of the negatively charged DA layer, and positively charged P-SS-T/PTX was adsorbed on the surface of drug-resistant tumor cells through electrostatic interactions to eventually target the nanoparticles to anchor to the mitochondrial outer membrane, resulting in depolarization of the mitochondrial membrane associated with a decrease in the mitochondrial membrane potential and an increase in mitochondrial membrane permeability. Therefore, a decrease in the expression of P-gp was induced by downregulation of mitochondrial ATP, and PTX released by disassembly of the nanoparticles directly diffused into the mitochondria, thus leading to mitochondrial DNA damage. Additionally, PTX released in the redox-responsive environment acted on the microtubules, inducing apoptosis of drug-resistant lung cancer cells. A combination of these effects induced apoptosis in drug-resistant tumor cells. Therefore, the present study provides a new strategy for the design and engineering of a smart nanoplatform for reversal of drug-resistance in tumors and inhibition of metastasis.

## Abbreviations

PTX: Paclitaxel
DA: Dimethylmaleic anhydride
TPP: Triphenylphosphonium
P85: Pluronic P85
DA-P-SS-T: DA-P85-SS-TPP; P-SS-T, P85-SS-TPP
GSH: Glutathione
MDR: Multidrug resistance
PF127: Pluronic F127
HA: Hyaluronic acid
DCC: N,N′-dicyclohexylcarbodiimide
DMAP: 4-dimethylaminopyridine
C6: Coumarin 6
H&E: Hematoxylin and eosin
DBU: 1,8-diazabicycloundec-7-ene
TFA: Trifluoroacetic acid
DLC: Drug loading capacity
DLE: Drug loading efficiency
CDDP: Cisplatin
TEM: Transmission electron microscopy
PBS: Phosphate-buffered saline
FBS: Fetal bovine serum
JC-1: 5,5',6,6'-tetrachloro-1,1',3,3'-tetraethylbenzimidazolcarbocyanine iodide
TIR: The tumor inhibitory rate
